# Associations between cMIND diet, mold exposure, and visual impairment among older adults in China: a national cross-sectional study

**DOI:** 10.3389/fnut.2026.1851210

**Published:** 2026-07-06

**Authors:** Jingshuang Qin, Xiaojie Pan, Chen Wu, Tongxu Zhang, Qing Yu, Min Li

**Affiliations:** 1 Department of Endocrinology and Metabolism, The First Hospital of China Medical University, Shenyang, Liaoning, China; 2 School of Nursing, China Medical University, Shenyang, Liaoning, China; 3 Department of Rehabilitation, Shengjing Hospital of China Medical University, Shenyang, Liaoning, China

**Keywords:** CLHLS, cMIND diet, mold exposure, older adults, visual impairment

## Abstract

**Background:**

Visual impairment poses a major challenge to healthy ageing, and its occurrence may be related to both dietary factors and the indoor environment. However, evidence regarding the associations of the Chinese version of the Mediterranean-Dietary Approaches to Stop Hypertension Intervention for Neurodegenerative Delay (cMIND) diet and mold exposure with visual impairment remains limited.

**Methods:**

This study included 14031 participants aged ≥ 65 years from the 2018 wave of the Chinese Longitudinal Healthy Longevity Survey (CLHLS). The cMIND diet score was calculated based on a validated questionnaire, and mold exposure was defined based on self-reported frequent perception of musty odors at home. Visual impairment was defined based on responses to a visual function assessment. We used multivariable logistic regression analyses to assess the independent and joint associations of the cMIND diet and mold exposure with visual impairment.

**Results:**

Among the participants, the median age was 85.00 years, and 35.97% had visual impairment. For every 1-point increase in the cMIND diet score, lower odds of visual impairment were observed (OR = 0.91, 95% CI: 0.88–0.94), whereas mold exposure was associated with higher odds (OR = 1.19, 95% CI: 1.04–1.35). A linear inverse association was observed between the cMIND diet score and visual impairment. Joint analysis showed the highest odds of visual impairment among participants with both low cMIND diet scores and mold exposure (OR = 1.68, 95% CI: 1.38–2.05). Findings were robust in sensitivity analyses.

**Conclusion:**

Independent and joint associations of the cMIND diet and mold exposure with visual impairment were observed among older adults, highlighting the potential importance of dietary and indoor environmental factors for visual health.

## Introduction

Population aging is occurring at an unprecedented pace worldwide, posing substantial challenges to health systems and societies (1). Promoting healthy aging has therefore become a major global public health priority (2). China, which has the largest population of older adults in the world, is experiencing a rapid demographic transition (3). Among the various health problems affecting Chinese older adults, visual impairment has emerged as an important public health concern (4, 5).

In 2020, an estimated 295 million individuals globally were affected by moderate to severe vision impairment, and an additional 43.3 million people were blind (6). In China, vision impairment ranks second among all causes of years lived with disability, following hearing impairment (7). Visual impairment in older adults not only affects daily life but is also associated with adverse outcomes such as falls (8), cognitive impairment (9), depression (10), social isolation (11), and reduced quality of life (12). Given that over 90% of visual impairments are preventable or treatable (13), identifying modifiable factors related to visual health has significant public health implications.

Dietary factors have therefore attracted increasing attention for their potential role in promoting visual health. Previous studies have linked specific nutrients to ocular health (14, 15), whereas dietary patterns may better reflect habitual diet and the combined effects of multiple foods. In this context, the Mediterranean-DASH Intervention for Neurodegenerative Delay (MIND) diet, which integrates key components of the Mediterranean diet and the Dietary Approaches to Stop Hypertension (DASH) diet, was originally developed to delay neurodegenerative decline (16). Given differences between Western and Chinese dietary habits, the culturally adapted Chinese version (cMIND) has been proposed to better reflect diet quality in older Chinese adults (17). Prior studies have suggested that higher adherence to the cMIND diet is associated with a lower risk of cognitive impairment in Chinese older adults (18), and this dietary pattern has also been applied to other aging-related health outcomes (19–22). These findings suggest that the cMIND diet may reflect a broader pattern of healthy aging-related dietary quality. Furthermore, considering that the retina and optic nerve are closely connected to the central nervous system (23, 24), this provides a theoretical basis for a potential association between the cMIND diet and visual health. As an overall dietary pattern characterized by food and nutrient profiles with potential antioxidant and anti-inflammatory effects, the cMIND diet may also be relevant to visual health, particularly given the roles of oxidative stress, chronic inflammation, and microvascular dysfunction in visual impairment (25, 26). However, quantitative evidence on the association between cMIND adherence and visual impairment in older adults remains limited.

In addition to dietary factors, environmental exposures may also be relevant to visual health. The World Health Organization (WHO) recognizes indoor mold exposure as an important environmental health concern, and residential mold contamination is common worldwide, with prevalence estimates ranging from 10 to 50% (27). Mold exposure, often reflected by the presence of musty odor, is commonly used in epidemiological studies as an indicator of indoor dampness and mold contamination (28, 29). Previous studies have suggested that mold exposure is associated with allergic reactions (30), respiratory diseases (31), arthritis (32), mental health problems (29, 33), and certain neurological symptoms (34), suggesting that its adverse health effects may be broad-ranging. However, epidemiological evidence regarding its association with visual outcomes remains limited, particularly among older adults. Older adults spend more time indoors and are therefore more likely to experience sustained indoor exposure, while domestic evidence regarding the association between mold exposure and visual impairment remains limited. These considerations highlight the importance of examining this association among older adults in China.

Recent epidemiological studies have increasingly examined dietary factors and environmental exposures in combination in relation to chronic health outcomes, such as chronic obstructive pulmonary disease and heart failure (35–37). Compared with evaluating either factor alone, joint assessment may provide a more comprehensive view of related health risks. Mechanistically, mold exposure may be associated with increased oxidative damage and inflammation (38), whereas higher adherence to the cMIND diet may indicate greater antioxidant and anti-inflammatory dietary potential (39). When dietary antioxidant and anti-inflammatory capacity is insufficient, the body may be less able to counter mold-related reactive oxygen species and inflammatory damage. Therefore, these mechanisms provide a stronger biological basis for jointly evaluating cMIND diet and mold exposure in relation to visual impairment. However, evidence on the joint association of cMIND diet and mold exposure with visual impairment in older adults remains limited.

To further investigate these relationships, this study used data from the Chinese Longitudinal Healthy Longevity Survey (CLHLS) to examine the independent and joint associations of cMIND diet and mold exposure with visual impairment among older adults in China.

## Materials and methods

### Study population

This study used data from the 2018 wave of the CLHLS. The CLHLS is a nationwide longitudinal healthy aging survey launched in 1998 and conducted every 2–3 years thereafter. It employs a multistage, cluster-random sampling method to recruit participants from 22 provinces across China. Detailed descriptions of the sampling methods can be found in previous literature (40, 41). The CLHLS data provides information on the socio-demographic characteristics, dietary behaviors, environmental conditions, and chronic diseases of older adults.

**FIGURE 1:**
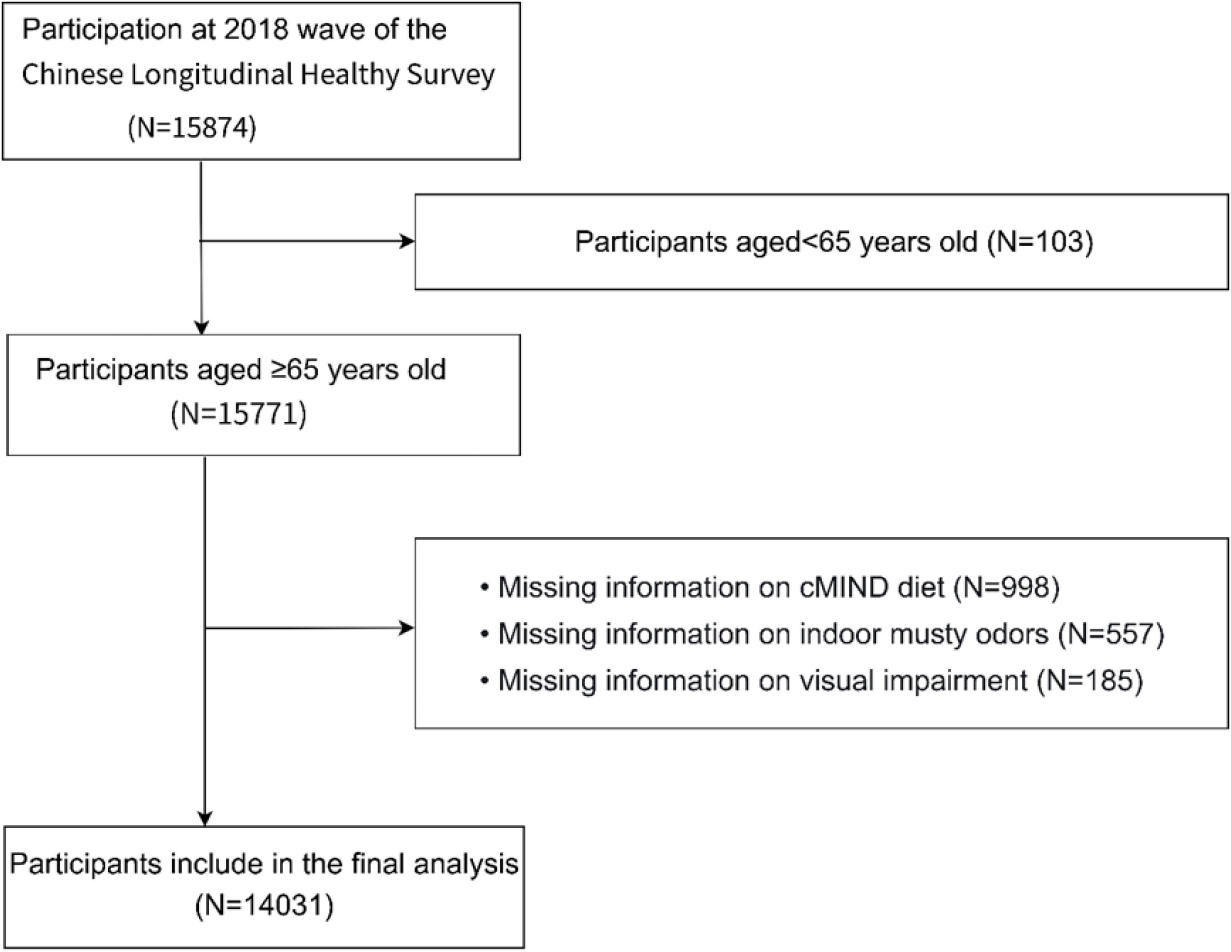
Flow chart of inclusion of participants in this study.

In the 2018 CLHLS wave, trained investigators collected information from 15,874 participants through face-to-face interviews. After excluding 103 participants aged under 65 years, 998 with missing information on cMIND diet, 557 with missing information on mold exposure, and 185 with missing or incomplete data on visual impairment, the final analytical sample comprised 14,031 individuals (Figure 1).

The CLHLS was approved by the Peking University Biomedical Ethics Committee (IRB00001052-13074). All participants provided written informed consent before taking part in the study.

### Assessment of cMIND diet

The cMIND questionnaire for older Chinese adults was developed and validated by Huang et al. using the MIND diet framework and CLHLS food frequency questionnaire (FFQ) data (17). The cMIND diet consists of 12 food groups: types of staple food (refined/whole grains), amount of staple food, fresh fruit, fresh vegetables, cooking oil (animal/ vegetable oil), mushrooms or algae, fish, food made from beans, nuts, garlic, tea, and white sugar or candy. Types of staple food, amount of staple food, and cooking oil, assign a score of 0 or 1; for all other items, assign a score of 0, 0.5, or 1. Further details regarding the scoring of the cMIND diet and the characteristics of the components are provided in Supplementary Tables 1, 2. The total score ranged from 0 to 12, with higher scores indicating greater adherence to the cMIND diet. The cMIND diet total score was divided into tertiles: low (0–4 points), medium (4.5–5.5 points), and high (6–12 points).

### Assessment of mold exposure

Assess mold exposure by asking the question, “Do you often smell musty odors at home?” Responses are dichotomized into “Yes” or “No.” The notion that musty odors can serve as a surrogate indicator for fungal exposure has been validated in previous literature (42).

### Assessment of visual impairment

According to previous CLHLS-based studies (43, 44), visual function in the CLHLS has been described as being assessed using an adapted Landolt-C chart. The circle on the visual acuity chart was illuminated with a flashlight, and participants were asked whether they could identify any break and its direction without wearing glasses. Four response options were provided: (1) Able to identify the break and its direction; (2) Able to identify the break but not its direction; (3) Unable to see the circle clearly; (4) Blind. Based on prior research (45, 46), respondents who could not identify the break and its direction were classified as having visual impairment.

### Covariates

Based on prior evidence (40, 47, 48), we adjusted for a range of potential confounders, encompassing participants’ sociodemographic characteristics, health-related behaviors, and health status. Sociodemographic characteristics included age (continuous), sex (female, male), area of residence (urban, rural), ethnicity (Han, others), marital status (have no spouse, have a spouse), and educational level (0 year, 1–6 years, ≥ 7 years). Health-related behaviors included smoking status (no, yes), alcohol consumption (no, yes), and physical activity (no, yes). Health status included hypertension (no, yes), diabetes (no, yes), heart disease (no, yes), and dementia (no, yes).

### Statistical analysis

Firstly, the distribution of continuous variables was tested using the Kolmogorov-Smirnov test and visual inspection of Q-Q plots, which indicated that the data were not normally distributed. Continuous variables were presented as median and the 25th and 75th percentiles (P_25_, P_75_), while categorical variables were reported as frequencies and percentages. Categorical variables were compared using the chi-square test, and continuous variables were compared using the Kruskal-Wallis H test, to evaluate differences in sociodemographic characteristics, health-related behaviors, and health status across cMIND dietary score tertiles (low, medium, and high). Secondly, multivariable binary logistic regression models were constructed to examine the independent and joint associations of the cMIND diet and mold exposure with visual impairment, and the results were presented as odds ratios (ORs) with corresponding 95% confidence intervals (CIs). Model 1 was unadjusted. Model 2 adjusted for age, sex, area of residence, ethnicity, marital status, and education level. Model 3 was further adjusted for smoking status, alcohol consumption, physical activity, hypertension, diabetes, heart disease, and dementia. Thirdly, restricted cubic spline (RCS) curves were fitted using knots placed at the 5th, 35th, 65th, and 95th percentiles of the cMIND diet score distribution to examine the potential non-linear association between the cMIND diet score and visual impairment. Fourth, stratified and interaction analyses were performed to assess potential effect modification in the associations of the cMIND diet and mold exposure with visual impairment. Analyses were stratified by sex, area of residence, marital status, education level, smoking status, alcohol consumption, physical activity, hypertension, diabetes, heart disease, and dementia.

Finally, two sensitivity analyses were conducted to assess the robustness of the findings. The first sensitivity analysis was performed using multiple imputation with chained equations to evaluate the potential impact of missing covariate data on the effect estimates. In the second sensitivity analysis, a total of 6,697 participants who had hypertension, diabetes, and heart disease were excluded. The data were analyzed using SPSS 27.0 and R 4.5.0, with statistical significance set at a two-sided *P* < 0.05.

## Results

### Study population characteristics

Table 1 presents the characteristics of participants according to tertiles of the cMIND diet score. The median age of the participants is 85.00 years, 56.25% are female, and the prevalence of visual impairment is 35.97%. Compared with participants with high cMIND diet scores, those with low and medium cMIND diet scores were older and less likely to live in urban areas, have higher education, or report mold exposure. All *P* < 0.001.

**TABLE 1:** Characteristics of the participants.

Characteristics	Total (*N* = 14,031)	Low (0–4) (*N* = 5,972)	Medium (4.5–5.5) (*N* = 4,720)	High (6–12) (*N* = 3,339)	*P*-value
Age, median (P_25_, P_75_)	85.00(76.00, 95.00)	89.00(80.00, 99.00)	84.00(75.00, 94.00)	80.00 (72.00, 90.00)	< 0.001
Sex, n (%)					< 0.001
Female	7,893 (56.25)	3,778 (63.26)	2,581 (54.68)	1,534 (45.94)	
Male	6,138 (43.75)	2,194 (36.74)	2,139 (45.32)	1,805 (54.06)	
Area of residence, n (%)					< 0.001
Urban	2,961 (21.10)	644 (10.78)	937 (19.85)	1,380 (41.33)	
Rural	11,070 (78.90)	5,328 (89.22)	3,783 (80.15)	1,959 (58.67)	
Ethnicity, n (%)					< 0.001
Han	11,380 (93.91)	4,652 (91.61)	3,845 (95.06)	2,883 (96.26)	
Others	738 (6.09)	426 (8.39)	200 (4.94)	112 (3.74)	
Marital status, n (%)					< 0.001
Have no spouse	8,065 (58.02)	4,029 (68.13)	2,625 (56.15)	1,411 (42.60)	
Have a spouse	5,836 (41.98)	1,885 (31.87)	2,050 (43.85)	1,901 (57.40)	
Education level, n (%)					< 0.001
0 year	6,045 (50.38)	3,236 (64.53)	1,938 (48.41)	871 (29.21)	
1–6 years	3,791 (31.59)	1,369 (27.30)	1,394 (34.82)	1,028 (34.47)	
≥7 years	2,164 (18.03)	410 (8.18)	671 (16.76)	1,083 (36.32)	
Smoking status, n (%)					< 0.001
No	11,829 (85.06)	5,125 (86.48)	3,926 (84.05)	2,778 (83.95)	
Yes	2,077 (14.94)	801 (13.52)	745 (15.95)	531 (16.05)	
Alcohol consumption, n (%)					< 0.001
No	11,852 (85.70)	5,216 (88.47)	3,953 (84.96)	2,683 (81.77)	
Yes	1,978 (14.30)	680 (11.53)	700 (15.04)	598 (18.23)	
Physical activity, n (%)					< 0.001
No	9,667 (69.84)	4,721 (80.26)	3,253 (69.76)	1,693 (51.35)	
Yes	4,175 (30.16)	1,161 (19.74)	1,410 (30.24)	1,604 (48.65)	
Hypertension, n (%)					< 0.001
No	7,518 (57.53)	3,416 (62.68)	2,501 (56.49)	1,601 (50.16)	
Yes	5,551 (42.47)	2,034 (37.32)	1,926 (43.51)	1,591 (49.84)	
Diabetes, n (%)					< 0.001
No	11,368 (90.11)	4,918 (93.66)	3,863 (90.09)	2,587 (84.08)	
Yes	1,248 (9.89)	333 (6.34)	425 (9.91)	490 (15.93)	
Heart disease, n (%)					< 0.001
No	10,470 (82.68)	4,483 (84.89)	3,606 (83.86)	2,381 (77.23)	
Yes	2,194 (17.32)	798 (15.11)	694 (16.14)	702 (22.77)	
Dementia, n (%)					< 0.001
No	12,262 (97.62)	5,091 (96.70)	4,174 (97.98)	2,997 (98.72)	
Yes	299 (2.38)	174 (3.31)	86 (2.019)	39 (1.29)	
Mold exposure, n (%)					< 0.001
No	12,072 (86.04)	4,936 (82.65)	4,111 (87.10)	3,025 (90.60)	
Yes	1,959 (13.96)	1,036 (17.35)	609 (12.90)	314 (9.40)	
Visual impairment, n (%)					< 0.001
No	8,984 (64.03)	3,252 (54.45)	3,137 (66.46)	2,595 (77.72)	
Yes	5,047 (35.97)	2,720 (45.55)	1,583 (33.54)	744 (22.28)	

Total percentages within categories may not equal 100% due to rounding.

### Association of the cMIND diet with visual impairment

**FIGURE 2:**
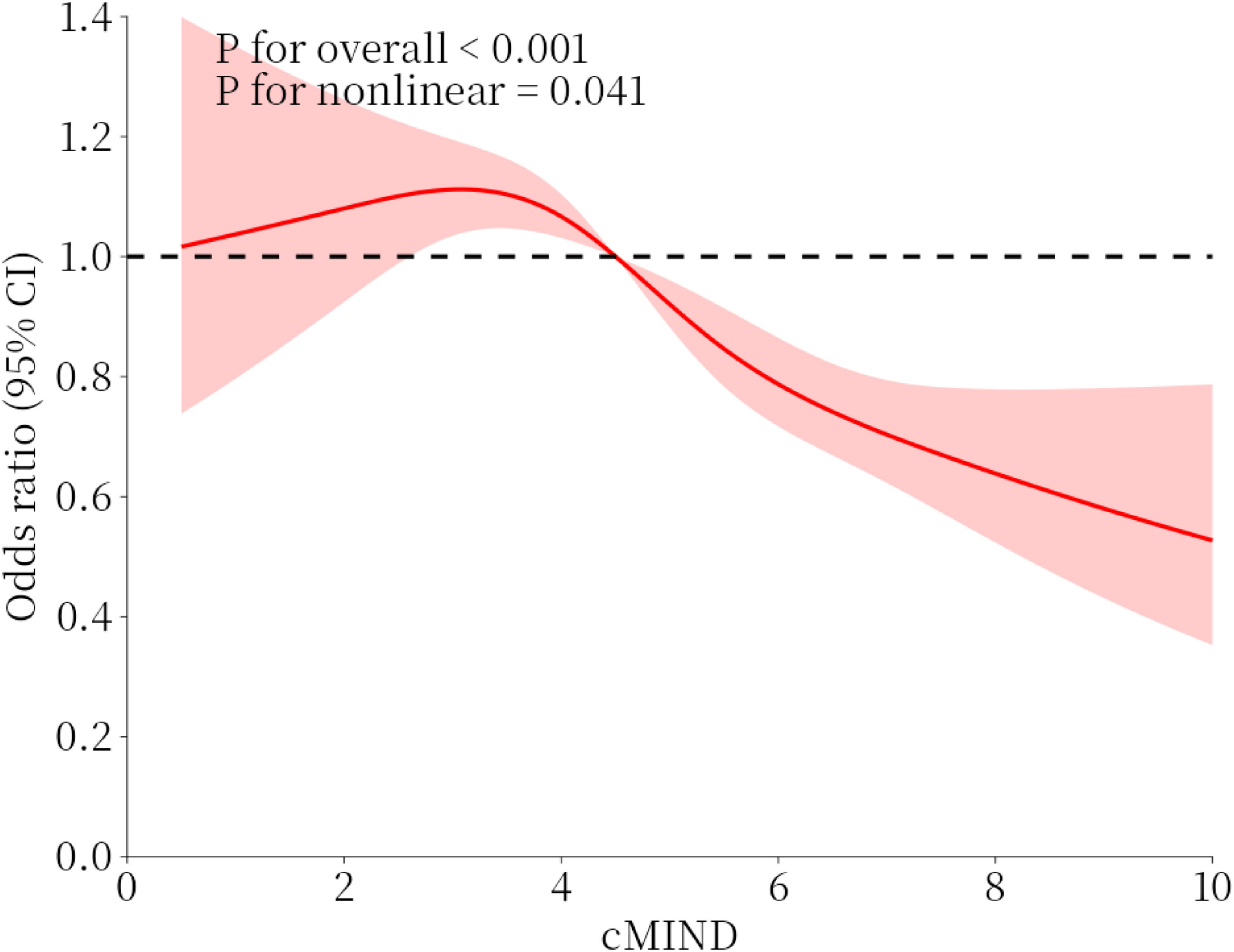
The association between cMIND diet and visual impairment using restricted cubic spline (RCS) regression analysis. Adjustments: age, sex, area of residence, ethnicity, marital status, education level, smoking status, alcohol consumption, physical activity, hypertension, diabetes, heart disease, and dementia.

Table 2 presents the association of the cMIND diet with visual impairment. After multivariable adjustment, for every 1-point increase in the cMIND diet score, lower odds of visual impairment were observed (OR = 0.91, 95% CI: 0.88–0.94). Compared with the low-adherence group, participants with medium and high adherence to the cMIND diet had lower odds of visual impairment (OR = 0.88, 95% CI: 0.79–0.98; OR = 0.64, 95% CI: 0.56–0.73, respectively). In the fully adjusted model, RCS analysis showed a significant association of the cMIND diet score with visual impairment (overall *P* < 0.001), with evidence of nonlinearity (P for nonlinearity = 0.041) The RCS curve suggested that the inverse association became more apparent when the cMIND score was above approximately 4.5 points (Figure 2).

**TABLE 2:** Association between cMIND diet and visual impairment.

Variables	Model 1		Model 2		Model 3	
	OR (95%CI)	*P*-value	OR (95%CI)	*P*-value	OR (95%CI)	*P*-value
cMIND diet was used as a continuous variable	0.75(0.74, 0.77)	< 0.001	0.87(0.85, 0.90)	< 0.001	0.91(0.88, 0.94)	< 0.001
cMIND diet was used as a categorical variable [vs. Low (0–4)]	–	–	–	–	–	–
Medium (4.5–5.5)	0.60(0.56, 0.65)	< 0.001	0.81(0.73, 0.89)	< 0.001	0.88(0.79, 0.98)	0.016
High (6–12)	0.34(0.31, 0.38)	< 0.001	0.57(0.51, 0.64)	< 0.001	0.64(0.56, 0.73)	< 0.001

OR, Odds ratios; CI, Confidence intervals. Model 1 was unadjusted. Model 2 was adjusted for age, sex, area of residence, ethnicity, marital status, and education level. Model 3 was adjusted for age, sex, area of residence, ethnicity, marital status, education level, smoking status, alcohol consumption, physical activity, hypertension, diabetes, heart disease, and dementia.

### Association of mold exposure with visual impairment

Table 3 presents the association between mold exposure and visual impairment. In Model 3, mold exposure was associated with a higher odds of visual impairment compared with no mold exposure (OR = 1.19, 95% CI: 1.04–1.35).

**TABLE 3:** Association between mold exposure and visual impairment.

Model	Had no mold exposure	Had mold exposure	*P*-value
	OR (95%CI)		
Model 1	Reference	1.26 (1.15, 1.39)	< 0.001
Model 2	Reference	1.17 (1.04, 1.32)	0.009
Model 3	Reference	1.19 (1.04, 1.35)	0.010

OR, Odds ratios; CI, Confidence intervals. Model 1 was unadjusted. Model 2 was adjusted for age, sex, area of residence, ethnicity, marital status, and education level. Model 3 was adjusted for age, sex, area of residence, ethnicity, marital status, education level, smoking status, alcohol consumption, physical activity, hypertension, diabetes, heart disease, and dementia.

### Joint associations of the cMIND diet and mold exposure with visual impairment

Table 4 summarizes the joint associations of the cMIND diet score and mold exposure with visual impairment. Compared with participants with a cMIND score of 6–12 and no mold exposure, lower cMIND scores were associated with a higher odds of visual impairment. Participants with both low cMIND scores (0–4) and mold exposure had the highest odds of visual impairment (OR = 1.68, 95% CI: 1.38–2.05).

**TABLE 4:** Joint effects of cMIND diet and mold exposure on visual impairment.

cMIND diet, score	Mold exposure	OR (95%CI)	*P*-value
0–4			
	Had no mold exposure	1.59 (1.38, 1.83)	< 0.001
	Had mold exposure	1.68 (1.38, 2.05)	< 0.001
4.5–5.5			
	Had no mold exposure	1.38 (1.19, 1.58)	< 0.001
	Had mold exposure	1.67 (1.31, 2.12)	< 0.001
6–12			
	Had no mold exposure	1.00	–
	Had mold exposure	1.39 (1.00, 1.94)	0.049

Adjusted for age, sex, area of residence, ethnicity, marital status, education level, smoking status, alcohol consumption, physical activity, hypertension, diabetes, heart disease, and dementia.

### Subgroup and interaction analyses

Interaction analyses were conducted to evaluate potential effect modification in the associations of the cMIND diet score and mold exposure with visual impairment. For the cMIND diet score, significant effect modification was observed by area of residence (P for interaction = 0.048) and alcohol consumption (P for interaction = 0.012). The inverse association was stronger among urban residents (OR = 0.83, 95% CI: 0.78–0.89) and participants without alcohol consumption (OR = 0.89, 95% CI: 0.86–0.93) (Supplementary Table 3). For mold exposure, significant effect modification was observed by area of residence (P for interaction < 0.001), education level (*P* for interaction < 0.001), and diabetes (*P* for interaction = 0.010). The positive association with visual impairment was stronger among urban residents (OR = 1.88, 95% CI: 1.37–2.59), participants with ≥ 7 years of education (OR = 2.08, 95% CI: 1.45–3.00), and those with diabetes (OR = 1.79, 95% CI: 1.19–2.67) (Supplementary Table 4). To evaluate subgroup-specific associations, stratified analyses of the joint associations of cMIND diet and mold exposure with visual impairment were conducted according to area of residence, alcohol consumption, education level, and diabetes status, using participants with high cMIND scores and no mold exposure as the reference group (Supplementary Tables 5–8). In the residence-stratified analysis, among urban participants, both participants with the low cMIND scores and mold exposure and those with the medium cMIND scores and mold exposure showed significantly higher odds of visual impairment (OR = 2.57, 95% CI: 1.47–4.51; OR = 3.16, 95% CI: 1.83–5.43, respectively), and the association appeared more pronounced among urban than rural participants. Among non-drinkers, participants with ≥ 7 years of education, and participants without diabetes, the highest odds of visual impairment were observed in the low cMIND scores with mold exposure group (OR = 1.87, 95% CI: 1.51–2.32; OR = 2.86, 95% CI: 1.45–5.62; OR = 1.66, 95% CI: 1.34–2.05, respectively).

### Sensitivity analyses

In the first sensitivity analysis, missing values were handled using multiple imputation, and the results remained stable (all *P* < 0.05) (Supplementary Tables 9, 10). In the second sensitivity analysis, participants with hypertension, diabetes, or heart disease were excluded, and the findings were consistent with those of the primary analysis (all *P* < 0.05) (Supplementary Table 11).

## Discussion

To our knowledge, this is the first study to investigate the independent and joint associations between the cMIND diet and mold exposure with visual impairment. We observed that higher cMIND diet scores were associated with lower odds of visual impairment, whereas mold exposure was associated with higher odds. Notably, joint analysis revealed that the highest odds were observed among participants with both low cMIND diet scores and mold exposure. These findings highlight a potential synergistic effect of dietary and environmental exposures on visual health among older adults.

Our results align with previous evidence indicating that diet is relevant to visual health. In Western populations, adherence to the Mediterranean diet is associated with slower visual decline and a lower risk or progression of age-related macular degeneration (49–51). The Rotterdam Study also found that higher adherence to the MIND diet was associated with a lower incidence of open-angle glaucoma (52). However, these dietary indices may not fully capture the dietary characteristics of Chinese older adults. A study among Chinese adults aged ≥ 80 years reported that higher dietary diversity scores (DDS) were associated with a lower risk of visual impairment (46). Because DDS mainly reflects overall dietary variety rather than specific healthy dietary patterns, this study further explored the association between adherence to the cMIND dietary pattern and visual impairment among Chinese older adults. Several mechanisms may partly explain the observed association. Carotenoids such as lutein and zeaxanthin found in leafy vegetables (53, 54), as well as ergothioneine present in mushrooms (55), may help protect retinal cells from oxidative stress (56, 57), a key pathological process underlying age-related visual decline. In addition, omega-3 fatty acids, particularly those derived from fish, may help reduce systemic inflammation and support retinal microvascular function (58, 59). Furthermore, because the retina and optic nerve are extensions of the central nervous system (60), dietary patterns originally developed to support brain health, such as the MIND diet and its adapted cMIND version (16), may also exert neuroprotective effects on visual pathways and help preserve visual function (52). Subgroup analyses further suggested that the association of adherence to the cMIND diet with visual impairment varied across certain population characteristics. The protective association was more pronounced among urban residents, who are exposed to higher levels of environmental oxidative stressors such as air pollution and light pollution (61), potentially increasing susceptibility to retinal damage (62, 63). Adherence to the cMIND diet may help reduce this vulnerability. Moreover, urban areas have better access to fresh foods and healthcare services, which helps improve adherence and early detection of visual impairment (64–66). In addition, the association was mainly observed among individuals who did not consume alcohol, whereas it was attenuated among those reporting alcohol consumption. This may be partly because alcohol intake can increase oxidative stress (67) and exert harmful effects on the retina (68). Moreover, alcohol consumption is often associated with other lifestyle factors, such as irregular sleep (69), and lower overall adherence to healthy behaviors (70), which could further diminish the protective effect of diet. Overall, these findings suggest that environmental and behavioral factors may modify the association of the cMIND diet with visual impairment.

Most existing studies have primarily focused on respiratory and allergic outcomes, while the potential visual effects of mold exposure have received relatively little attention. For example, several studies have linked mold exposure to eye-related symptoms, including dryness, irritation, and discomfort (71, 72). In addition, environmental molds such as *Aspergillus* and *Fusarium* are also well-recognized causes of fungal keratitis, a severe corneal infection that can lead to corneal damage and visual impairment (73, 74). Some evidence has also suggested that mold exposure may be linked to inflammatory chorioretinal disorders such as multifocal choroiditis, which can impair visual function (75). These findings suggest that mold exposure may affect multiple ocular structures and potentially contribute to visual impairment. However, epidemiological evidence directly linking mold exposure to visual impairment remains scarce. Several biological mechanisms may explain this association. Molds can produce mycotoxins and microbial volatile organic compounds that may induce inflammatory responses and oxidative stress in ocular tissues (73, 76). In addition, chronic exposure to mold spores may trigger immune reactions and ocular surface inflammation, potentially affecting corneal integrity and visual function (77). Older adults may be particularly susceptible to these effects because of age-related ocular degeneration and reduced antioxidant capacity, which may further increase the risk of visual impairment (78). Additionally, interaction analysis revealed that area of residence, education level, and diabetes status might influence the association between mold exposure and visual impairment. Stronger associations were observed among urban residents, participants with ≥ 7 years of education, and those with diabetes. For urban older adults, this may be partly explained by residential characteristics such as higher housing density, older infrastructure, and limited ventilation (79, 80), which can increase cumulative indoor mold exposure, particularly given their prolonged indoor residence. In addition, older adults with diabetes may be more vulnerable to the visual effects of mold exposure because chronic hyperglycemia can induce oxidative stress and inflammatory responses, which contribute to microvascular dysfunction and increase susceptibility to visual impairment (81). Individuals with ≥ 7 years of education may have greater health awareness and access to information, which could make them more sensitive to environmental risks such as mold exposure (82, 83).

One finding worth noting is the clear gradient observed in the joint analysis of cMIND diet and mold exposure. Compared to those with high cMIND diet scores and no mold exposure, older adults with medium or low cMIND diet scores had higher odds of visual impairment, with the highest odds observed in those who had both mold exposure and low cMIND diet scores. This pattern suggests that dietary and indoor environmental factors may jointly influence visual health in older adults. Low adherence to the cMIND diet results in insufficient intake of protective nutrients, thereby impairing antioxidant and anti-inflammatory defenses and potentiating mold exposure-induced oxidative stress and inflammatory responses (84, 85). Specifically, diminished antioxidant capacity may reduce the clearance of reactive oxygen species generated in response to mold (86, 87), while mold-induced pro-inflammatory cytokines further compromise retinal neurovascular function (87–89). From a practical perspective, promoting healthier dietary patterns, together with efforts to reduce household dampness and mold exposure, may represent complementary approaches to support visual health among older adults.

Several limitations in this study should be acknowledged. Firstly, given the cross-sectional design, causal inferences cannot be made. Secondly, information on mold exposure and the cMIND diet was based on self-reported data, which may have introduced recall bias. In addition, as the CLHLS is a large-scale population-based survey, visual impairment was identified using a visual function assessment rather than a clinical ophthalmologic diagnosis. The assessment was based on uncorrected visual function and therefore may not fully distinguish refractive error from pathological visual impairment. Thirdly, although multiple potential confounders were adjusted for, some relevant factors, such as detailed ophthalmologic conditions and indoor environmental characteristics, were not available in the CLHLS dataset.

## Conclusion

Based on a nationally representative sample of Chinese older adults, this study provides new epidemiological evidence on the independent and joint associations of the cMIND diet and mold exposure with visual impairment. Higher cMIND diet scores were associated with lower odds of visual impairment, while older adults with low cMIND diet scores combined with mold exposure had the highest odds. Stratified joint analyses indicated that urban residents are particularly vulnerable to this combined effect, indicating that this group belongs to a high-risk population. Public health strategies targeting this population (such as improving diet and reducing indoor mold exposure) may help prevent visual impairment. Together, these results indicate that visual impairment may be influenced by multiple modifiable factors rather than a single exposure alone, which has important implications for visual health and healthy aging.

## Data Availability

Publicly available datasets were analyzed in this study. This data can be found at: https://opendata.pku.edu.cn/dataverse/CHADS.

## References

[1] Khan H Addo K Findlay H. *Public health chall*enges and responses to the growing ageing populations. *Public Health Chall.* (2024) 3:e213. 10.1002/puh2.213 40496520 PMC12039680

[2] The Lancet Healthy Longevity. The decade of healthy ageing: progress and challenges ahead. *Lancet Healthy Longev.* (2024) 5:e1. 10.1016/S2666-7568(23)00271-4 38183990

[3] Chen X Giles J Yao Y Yip W Meng Q Berkman L et al. The path to healthy ageing in China: a Peking University-*Lancet* commission. *Lancet.* (2022) 400:1967–2006. 10.1016/S0140-6736(22)01546-X 36423650 PMC9801271

[4] Chen J Zhang S Yang F Jiang Y Lu Y Tang Y. Prevalence and causes of vision impairment in elderly Chinese people living in suburban Shanghai. *Asia Pac J Ophthalmol.* (2024) 13:100002. 10.1016/j.apjo.2023.100002 38383074

[5] Chen J Zhu Y Li Z Chen X Chen X Xie R et al. Temporal trends and projection of blindness and vision loss prevalence in older adults in BRICS countries. *J Am Geriatr Soc.* (2024) 72:544–50. 10.1111/jgs.18672 37960928

[6] GBD 2019 Blindness and Vision Impairment Collaborators, Vision Loss Expert Group of the Global Burden of Disease Study. Trends in prevalence of blindness and distance and near vision impairment over 30 years: an analysis for the Global Burden of Disease Study. *Lancet Glob Health.* (2021) 9:e130–43. 10.1016/S2214-109X(20)30425-3 33275950 PMC7820390

[7] Xu T Wang B Liu H Wang H Yin P Dong W et al. Prevalence and causes of vision loss in China from 1990 to 2019: findings from the Global Burden of Disease Study 2019. *Lancet Public Health.* (2020) 5:e682–91. 10.1016/S2468-2667(20)30254-1 33271081

[8] Thomas J Almidani L Ramulu P Varadaraj V. Falls and multiple falls among united states older adults with vision impairment. *Am J Ophthalmol.* (2025) 271:166–74. 10.1016/j.ajo.2024.11.012 39603313

[9] Rahmati M Smith L Lee H Boyer L Fond G Yon D et al. Associations between vision impairment and eye diseases with dementia, dementia subtypes and cognitive impairment: an umbrella review. *Ageing Res Rev.* (2024) 101:102523. 10.1016/j.arr.2024.102523 39369799

[10] Zhao Z Wang S Congdon N Ma X. Self-reported vision impairment and depressive symptoms among older adults: a longitudinal mediation analysis. *Br J Ophthalmol.* (2025) 109:1433–8. 10.1136/bjo-2024-326072 40639924

[11] Trujillo Tanner C Yorgason J Richardson S Redelfs A Serrao Hill M White A et al. Sensory disabilities and social isolation among hispanic older adults: toward culturally sensitive measurement of social isolation. *J Gerontol B Psychol Sci Soc Sci.* (2022) 77:2091–100. 10.1093/geronb/gbac001 35022736 PMC9683500

[12] Man R Gan A Fenwick E Teo K Tan A Cheung G et al. Impact of incident age-related macular degeneration and associated vision loss on vision-related quality of life. *Br J Ophthalmol.* (2022) 106:1063–8. 10.1136/bjophthalmol-2020-318269 33637622

[13] Burton M Ramke J Marques A Bourne R Congdon N Jones I et al. The lancet global health commission on global eye health: vision beyond 2020. *Lancet Glob Health.* (2021) 9:e489–551. 10.1016/S2214-109X(20)30488-5 33607016 PMC7966694

[14] Ozawa Y Nagai N Suzuki M Kurihara T Shinoda H Watanabe M et al. [Effects of constant intake of lutein-rich spinach on macular pigment optical density: a pilot study]. *Nippon Ganka Gakkai Zasshi* (2016) 120:41–8. 26950968

[15] Rowan S Jiang S Chang M Volkin J Cassalman C Smith K et al. A low glycemic diet protects disease-prone Nrf2-deficient mice against age-related macular degeneration. *Free Radic Biol Med.* (2020) 150:75–86. 10.1016/j.freeradbiomed.2020.02.010 32068111 PMC7747150

[16] Morris M Tangney C Wang Y Sacks F Barnes L Bennett D et al. diet slows cognitive decline with aging. *Alzheimers Dement.* (2015) 11:1015–22. 10.1016/j.jalz.2015.04.011 26086182 PMC4581900

[17] Huang X Aihemaitijiang S Ye C Halimulati M Wang R Zhang Z. Development of the cMIND diet and its association with cognitive impairment in older Chinese people. *J Nutr Health Aging.* (2022) 26:760–70. 10.1007/s12603-022-1829-1 35934820 PMC12280751

[18] Cai D Zeng Y Xu X Ye M Song A Chen M. Association of the cMIND diet with cognitive impairment in older adults: evidence from a 10-year nationwide study. *Front Nutr.* (2025) 12:1716435. 10.3389/fnut.2025.1716435 41608510 PMC12834752

[19] Qi Y Xue X Chen N Gong J Mu D Zhao K et al. Adherence to the cMIND and AIDD diets and their associations with anxiety in older adults in China. *Front Nutr.* (2025) 12:1548072. 10.3389/fnut.2025.1548072 40098739 PMC11911210

[20] Yang L Li M Shu J Cao L. Association between cMIND diet adherence and frailty among Chinese older adults: A 10-year longitudinal study. *J Nutr Health Aging.* (2025) 29:100709. 10.1016/j.jnha.2025.100709 41108817 PMC12556186

[21] Dai Z Wu Y Chen Y Chen J Jiang H Huang H et al. Associations of chinese-modified MIND diet with low muscle mass and physical performance among old adults in china: findings from the CLHLS 2018 national survey. *Aging Clin Exp Res.* (2026) 38:75. 10.1007/s40520-026-03325-3 41627623 PMC12886234

[22] Xian X Cao S Wang X Zhang Z Yang X Zeng L et al. Sex differences in association of cMIND diet with all-cause mortality in Chinese older adults: a 10-year national cohort study. *BMC Public Health.* (2026) 26:782. 10.1186/s12889-026-26311-w 41629861 PMC12958577

[23] Marchesi N Fahmideh F Boschi F Pascale A Barbieri A. Ocular neurodegenerative diseases: interconnection between retina and cortical areas. *Cells.* (2021) 10:2394. 10.3390/cells10092394 34572041 PMC8469605

[24] Sharif N. Degeneration of retina-brain components and connections in glaucoma: disease causation and treatment options for eyesight preservation. *Curr Res Neurobiol.* (2022) 3:100037. 10.1016/j.crneur.2022.100037 36685768 PMC9846481

[25] Ruan Y Jiang S Musayeva A Gericke A. Oxidative stress and vascular dysfunction in the retina: therapeutic strategies. *Antioxidants.* (2020) 9:761. 10.3390/antiox9080761 32824523 PMC7465265

[26] Shi X Li P Liu H Prokosch V. Oxidative stress, vascular endothelium, and the pathology of neurodegeneration in retina. *Antioxidants.* (2022) 11:543. 10.3390/antiox11030543 35326193 PMC8944517

[27] World Health Organization. *WHO Guidelines Approved by the Guidelines Review Committee, in WHO Guidelines for Indoor Air Quality: Dampness and Mould.* Geneva: World Health Organization (2009). 23785740

[28] Ma X Zhao H Wang Y Hou M Liu W Sun M. Association of mold exposure and solid household fuel use with depression and anxiety among older adults in China. *Environ Health.* (2025) 24:50. 10.1186/s12940-025-01193-4 40696404 PMC12285112

[29] Xian X Fu Y Cao S Niu T. Association between mold exposure and depressive symptoms among Chinese older adults: results from the Chinese longitudinal healthy longevity survey (CLHLS). *J Affect Disord.* (2025) 386:119442. 10.1016/j.jad.2025.119442 40404105

[30] Wang J Janson C Malinovschi A Holm M Franklin K Modig L et al. Asthma, allergic rhinitis and atopic dermatitis in association with home environment - The RHINE study. *Sci Total Environ.* (2022) 853:158609. 10.1016/j.scitotenv.2022.158609 36089044

[31] Barnes C Khurana Hershey G. Indoor and outdoor fungal allergens and impacts on respiratory allergic disease. *J Allergy Clin Immunol Pract.* (2025) 13:1267–71. 10.1016/j.jaip.2025.03.023 40147627 PMC12483281

[32] Yu G Jiang K Chen W Tan T Cong L. Household mold exposure and arthritis in older Chinese adults: evidence from the Chinese longitudinal healthy longevity survey (CLHLS). *BMC Geriatr.* (2026) 26:293. 10.1186/s12877-026-07096-4 41634594 PMC12954978

[33] Zhang Z Xu H Zhou J Cao X. The impact of mold exposure on anxiety symptoms in the older adults: A moderated mediation model based on CLHLS. *Ecotoxicol Environ Saf.* (2024) 284:116967. 10.1016/j.ecoenv.2024.116967 39241605

[34] Liu X Sun X Wang X Xu J Zang S. Association between indoor musty odors and cognitive impairment among older adults. *Sci Rep.* (2025) 15:31943. 10.1038/s41598-025-12000-y 40883342 PMC12397269

[35] Wang T Li J Liang Y Han W Tang J Cheng G et al. Joint effects of carbon black exposure and dietary antioxidant vitamin intake on small airway dysfunction. *Front Nutr.* (2021) 8:716398. 10.3389/fnut.2021.716398 34760908 PMC8572798

[36] Ye E Xu Z Hou X Wang Y Zhou C Xiang J et al. Joint effects of air pollution and diet patterns on the risk of chronic obstructive pulmonary disease. *Sci Rep.* (2025) 15:13939. 10.1038/s41598-025-96603-5 40263431 PMC12015326

[37] Zhu S Zhang X Wu Z Jin Y Wu W Zhang J et al. Healthful plant-based dietary patterns, PM2⋅5 exposure and the risk of heart failure: a population-based cohort study. *Br J Nutr.* (2025) 133:1235–40. 10.1017/S0007114525000698 40181603

[38] Ehsanifar M Rajati R Gholami A Reiss J. Mold and mycotoxin exposure and brain disorders. *J Integr Neurosci.* (2023) 22:137. 10.31083/j.jin2206137 38176924

[39] Atabılen B Acar Özen P Tuncer A Polat MB Pinar A Akdevelıoğlu Y. Effect of MIND diet on oxidative stress markers in multiple sclerosis. *Mult Scler Relat Disord*. (2025) 101:106598. 10.1016/j.msard.2025.106598 40609490

[40] Wang X Yin Z Gao Q Song Y Xu H Zang S. Associations of indoor musty odors with depression and anxiety symptoms in Chinese older adults: a nationwide study. *BMC Public Health.* (2025) 25:2793. 10.1186/s12889-025-24096-y 40819072 PMC12357380

[41] Yu B Steptoe A Chen Y. Social isolation, loneliness, and all-cause mortality: a cohort study of 35,254 Chinese older adults. *J Am Geriatr Soc.* (2022) 70:1717–25. 10.1111/jgs.17708 35229887

[42] Sharpe R Thornton C Tyrrell J Nikolaou V Osborne N. Variable risk of atopic disease due to indoor fungal exposure in NHANES 2005-2006. *Clin Exp Allergy.* (2015) 45:1566–78. 10.1111/cea.12549 25845975

[43] Luo Y Zhang Q Han L Shen Z Chen Y Wang K et al. Trends in the prevalence of vision impairment among the oldest-old Chinese population from 1998 to 2018. *J Glob Health.* (2022) 12:11006. 10.7189/jogh.12.11006 35862489

[44] Huang A Zhang D Zhang L Zhou Z. Predictors and consequences of visual trajectories in Chinese older population: a growth mixture model. *J Glob Health.* (2024) 14:04080. 10.7189/jogh.14.04080 38817127 PMC11140284

[45] Zhang Y Ge M Zhao W Liu Y Xia X Hou L et al. Sensory impairment and all-cause mortality among the oldest-old: findings from the Chinese longitudinal healthy longevity survey (CLHLS). *J Nutr Health Aging.* (2020) 24:132–7. 10.1007/s12603-020-1319-2 32003401 PMC12879221

[46] Shen X Chen X Chen X Li Z Lin J Huang H et al. Association of vision and hearing impairment and dietary diversity among the oldest old in China: findings from the Chinese longitudinal healthy longevity survey. *BMC Public Health.* (2024) 24:1997. 10.1186/s12889-024-19482-x 39060927 PMC11282864

[47] Zhang Y Lan Y Mou Y Deng Y Chen Z Fu Y et al. Association between cMIND diet and dementia among Chinese older adults: a population-based cross-sectional study. *Nutrients.* (2025) 17:3529. 10.3390/nu17223529 41305579 PMC12655610

[48] Zhou L Huang H Wang Q Peng L. Association of visual and hearing impairments with all-cause mortality in older adults in China. *BMC Geriatr.* (2025) 25:572. 10.1186/s12877-025-06205-z 40745270 PMC12315374

[49] Merle B Colijn J Cougnard-Grégoire A de Koning-Backus A Delyfer M Kiefte-de Jong J et al. Mediterranean diet and incidence of advanced age-related macular degeneration: the EYE-RISK consortium. *Ophthalmology.* (2019) 126:381–90. 10.1016/j.ophtha.2018.08.006 30114418

[50] Merle B Rosner B Seddon J. Genetic susceptibility, diet quality, and two-step progression in drusen size. *Invest Ophthalmol Vis Sci.* (2020) 61:17. 10.1167/iovs.61.5.17 32407518 PMC7405620

[51] Agrón E Vance E Domalpally A Chew E Keenan T. Relationships between diet and geographic atrophy progression in the age-related eye diseases studies 1 and 2. *Nutrients.* (2025) 17:771. 10.3390/nu17050771 40077641 PMC11901604

[52] Vergroesen J de Crom T van Duijn C Voortman T Klaver C Ramdas WD. MIND diet lowers risk of open-angle glaucoma: the rotterdam study. *Eur J Nutr.* (2023) 62:477–87. 10.1007/s00394-022-03003-w 36123555 PMC9899739

[53] Ganeshbabu M Manochkumar J Efferth T Ramamoorthy S. Lutein: a natural defence combating age-related macular degeneration. *Phytomedicine.* (2025) 143:156578. 10.1016/j.phymed.2025.156578 40446575

[54] Lin M Lin C Chiu W Chang H Cheang W Chang C et al. Protective effects of microalgal macular pigment on diabetic retinopathy upon blue light irradiation induced oxidative stress, inflammation, and MAPK pathways. *Food Res Int.* (2025) 219:116978. 10.1016/j.foodres.2025.116978 40922204

[55] Kalaras M Richie J Calcagnotto A Beelman R. Mushrooms: a rich source of the antioxidants ergothioneine and glutathione. *Food Chem.* (2017) 233:429–33. 10.1016/j.foodchem.2017.04.109 28530594

[56] He W Tang P Lv H. Targeting oxidative stress in diabetic retinopathy: mechanisms, pathology, and novel treatment approaches. *Front Immunol.* (2025) 16:1571576. 10.3389/fimmu.2025.1571576 40589740 PMC12207546

[57] Wazny V Grgic J Valenzuela P Feng L Maier A. Ergothioneine and exercise: a match made in (cognitive) heaven? *Ageing Res Rev.* (2026) 114:102993. 10.1016/j.arr.2025.102993 41390100

[58] SanGiovanni J Chew E. The role of omega-3 long-chain polyunsaturated fatty acids in health and disease of the retina. *Prog Retin Eye Res.* (2005) 24:87–138. 10.1016/j.preteyeres.2004.06.002 15555528

[59] Shahidi F Ambigaipalan P. Omega-3 polyunsaturated fatty acids and their health benefits. *Annu Rev Food Sci Technol.* (2018) 9:345–81. 10.1146/annurev-food-111317-095850 29350557

[60] London A Benhar I Schwartz M. The retina as a window to the brain-from eye research to CNS disorders. *Nat Rev Neurol.* (2013) 9:44–53. 10.1038/nrneurol.2012.227 23165340

[61] Guillien A Welten M Amine I Montazeri P Oftedal B Tarhini Z et al. Lifetime urban exposure, socioeconomic position and respiratory health among adolescents. *Environ Res.* (2026) 303:124771. 10.1016/j.envres.2026.124771 42144213

[62] Lasagni Vitar R Hvozda Arana A Janezic N Marchini T Tau J Martinefski M et al. Urban air pollution induces redox imbalance and epithelium hyperplasia in mice cornea. *Toxicol Appl Pharmacol.* (2019) 384:114770. 10.1016/j.taap.2019.114770 31628919

[63] Chua S Khawaja A Dick A Morgan J Dhillon B Lotery A et al. Ambient air pollution associations with retinal morphology in the UK Biobank. *Invest Ophthalmol Vis Sci.* (2020) 61:32. 10.1167/iovs.61.5.32 32428233 PMC7405693

[64] Zhao D Zhou Z Shen C Nawaz R Li D Ren Y et al. Rural and urban differences in patient experience in China: a coarsened exact matching study from the perspective of residents. *BMC Health Serv Res.* (2021) 21:330. 10.1186/s12913-021-06328-0 33849544 PMC8042990

[65] Zhao J Zuo L Sun J Su C Wang H. Trends and Urban-rural disparities of energy intake and macronutrient composition among Chinese children: findings from the China health and nutrition survey (1991 to 2015). *Nutrients.* (2021) 13:1933. 10.3390/nu13061933 34199924 PMC8229111

[66] Fan H Xu Q Wang J Du M. Does the digital economy promote dietary diversity among chinese residents? *Foods.* (2025) 14:3873. 10.3390/foods14223873 41300031 PMC12650907

[67] Tsermpini E Plemenitaš Ilješ A Dolžan V. Alcohol-induced oxidative stress and the role of antioxidants in alcohol use disorder: a systematic review. *Antioxidants.* (2022) 11:1374. 10.3390/antiox11071374 35883865 PMC9311529

[68] Kleerekooper I Chua S Foster P Trip S Plant G Petzold A et al. Associations of alcohol consumption and smoking with disease risk and neurodegeneration in individuals with multiple sclerosis in the United Kingdom. *JAMA Netw Open.* (2022) 5:e220902. 10.1001/jamanetworkopen.2022.0902 35238934 PMC8895260

[69] Zheng D Yuan X Ma C Liu Y VanEvery H Sun Y et al. Alcohol consumption and sleep quality: a community-based study. *Public Health Nutr.* (2021) 24:4851–8. 10.1017/S1368980020004553 33183388 PMC11077453

[70] Skrzynski C Chen M Bryan A. More frequent solitary alcohol consumption is associated with poorer diet quality, worse sleep, higher body mass index, and more problematic alcohol use. *Ann Behav Med.* (2024) 58:763–7. 10.1093/abm/kaae046 39158936

[71] Zhang X Norbäck D Fan Q Bai X Li T Zhang Y et al. Dampness and mold in homes across China: associations with rhinitis, ocular, throat and dermal symptoms, headache and fatigue among adults. *Indoor Air.* (2019) 29:30–42. 10.1111/ina.12517 30379348

[72] Yang Q Wang J Norbäck D. The home environment in a nationwide sample of multi-family buildings in Sweden: associations with ocular, nasal, throat and dermal symptoms, headache, and fatigue among adults. *Indoor Air.* (2021) 31:1402–16. 10.1111/ina.12787 33682978

[73] Bossou Y Serssar Y Allou A Vitry S Momas I Seta N et al. Impact of mycotoxins secreted by aspergillus molds on the inflammatory response of human corneal epithelial cells. *Toxins.* (2017) 9:197. 10.3390/toxins9070197 28640227 PMC5535144

[74] Ghenciu L Faur A Bolintineanu S Salavat M Maghiari A. Recent advances in diagnosis and treatment approaches in fungal keratitis: a narrative review. *Microorganisms.* (2024) 12:161. 10.3390/microorganisms12010161 38257986 PMC10820712

[75] Rudich R Santilli J Rockwell W. Indoor mold spore exposure: a possible factor in the etiology of multifocal choroiditis. *Am J Ophthalmol.* (2003) 135:402–4. 10.1016/s0002-9394(02)01962-1 12614769

[76] Bennett J Inamdar A. Are some fungal volatile organic compounds (VOCs) mycotoxins? *Toxins.* (2015) 7:3785–804. 10.3390/toxins7093785 26402705 PMC4591661

[77] Abbondante S Leal S Clark H Ratitong B Sun Y Ma L et al. Immunity to pathogenic fungi in the eye. *Semin Immunol.* (2023) 67:101753. 10.1016/j.smim.2023.101753 37060806 PMC10508057

[78] You L Lin Y Zheng Y Han Z Zeng L Chen H. The impact of aging on ocular diseases: unveiling complex interactions. *Aging Dis.* (2024) 16:2803–30. 10.14336/AD.2024.0850 39500360 PMC12339180

[79] Norbäck D Zhang X Fan Q Zhang Z Zhang Y Li B et al. Home environment and health: domestic risk factors for rhinitis, throat symptoms and non-respiratory symptoms among adults across China. *Sci Total Environ.* (2019) 681:320–30. 10.1016/j.scitotenv.2019.05.084 31121396

[80] Hurraß J Nowak D Heinzow B Joest M Stemler J Wiesmüller G. Indoor mold—important considerations for medical advice to patients. *Dtsch Arztebl Int.* (2024) 121:265–71. 10.3238/arztebl.m2024.0018 38381662 PMC11381209

[81] Yang T Qi F Guo F Shao M Song Y Ren G et al. An update on chronic complications of diabetes mellitus: from molecular mechanisms to therapeutic strategies with a focus on metabolic memory. *Mol Med.* (2024) 30:71. 10.1186/s10020-024-00824-9 38797859 PMC11128119

[82] Carducci A Fiore M Azara A Bonaccorsi G Bortoletto M Caggiano G et al. Environment and health: Risk perception and its determinants among Italian university students. *Sci Total Environ.* (2019) 691:1162–72. 10.1016/j.scitotenv.2019.07.201 31466198

[83] Wei Z Zhang Z Guo L Zhou W Yang K. Positive relationship between education level and risk perception and behavioral response: a machine learning approach. *PLoS One.* (2025) 20:e0321153. 10.1371/journal.pone.0321153 40179062 PMC11967964

[84] Safaei M Kheirouri S Alizadeh M Pirovi A. Association between Mediterranean-dietary approaches to stop hypertension intervention for neurodegenerative delay diet and biomarkers of oxidative stress, metabolic factors, disease severity, and odds of disease in rheumatoid arthritis patients. *Food Sci Nutr.* (2024) 12:3973–81. 10.1002/fsn3.4055 38873478 PMC11167176

[85] Mózes N Varga J Szwajgier D Kryczyk-Poprawa A Zábó V Lehoczki A et al. Dietary polyphenols in brain aging: molecular mechanisms and implications for neurodegeneration. *Nutrients.* (2026) 18:1470. 10.3390/nu18091470 42124071 PMC13165310

[86] Pawlowska E Szczepanska J Koskela A Kaarniranta K Blasiak J. Dietary polyphenols in age-related macular degeneration: protection against oxidative stress and beyond. *Oxid Med Cell Longev.* (2019) 2019:9682318. 10.1155/2019/9682318 31019656 PMC6451822

[87] Macedo G Vieira P Rodrigues N Gomes K Rodrigues J Franco J et al. Effect of fungal indoor air pollutant 1-octen-3-ol on levels of reactive oxygen species and nitric oxide as well as dehydrogenases activities in drosophila melanogaster males. *J Toxicol Environ Health A.* (2022) 85:573–85. 10.1080/15287394.2022.2054887 35354383

[88] Øya E Afanou A Malla N Uhlig S Rolen E Skaar I et al. Characterization and pro-inflammatory responses of spore and hyphae samples from various mold species. *Indoor Air.* (2018) 28:28–39. 10.1111/ina.12426 28922584

[89] Ferreira L Williams K Best G Haydinger C Smith J. Inflammatory cytokines as mediators of retinal endothelial barrier dysfunction in non-infectious uveitis. *Clin Transl Immunol.* (2023) 12:e1479. 10.1002/cti2.1479 38090668 PMC10714664

