# Supplementary material for: Associations between cMIND diet, mold exposure, and visual impairment among older adults in China: a national cross-sectional study
Source: Front Nutr. 2026 Jul 6;13:1851210. doi: 10.3389/fnut.2026.1851210 (PMC13381192; doi:10.3389/fnut.2026.1851210)
Supplement: Supplementary file 1 [file Table_1.docx]

**Supplementary Table 1** Components and scores of cMIND diet

| Components | Score | | |
| --- | --- | --- | --- |
|  | 0 | 0.5 | 1 |
| Types of staple food | Refined grains |  | Whole grains |
| Amount of staple food | <250g or >400g |  | 250g-400g |
| Fresh fruit | ≤2 servings/week | 3-5/week | ≥6 servings/week |
| Fresh vegetables | ≤2 servings/week | 3-5/week | ≥6 servings/week |
| Cooking oil | Animal oil |  | Vegetable oil |
| Mushroom or algae | ≤1 meal/week | 1-3/week | ≥4 meals/week |
| Fish | <1/month | 1-3/month | ≥1 meal/week |
| Food made from beans | <1 meal/week | 1-3/week | ≥4 meals/week |
| Nut | <1 serving/week | 1-4/week | ≥5 servings/week |
| Garlic | <1 meal/week | 1-3/week | ≥4 meals/week |
| Tea | Not almost every day | Other types of tea (almost every day) | Green tea(almost every day) |
| White sugar or candy | ≥2 servings/week | 1/month-1/week | <1 serving/month |
