# Supplementary material for: Associations between cMIND diet, mold exposure, and visual impairment among older adults in China: a national cross-sectional study
Source: Front Nutr. 2026 Jul 6;13:1851210. doi: 10.3389/fnut.2026.1851210 (PMC13381192; doi:10.3389/fnut.2026.1851210)
Supplement: Supplementary file 2 [file Table_2.docx]

**Supplementary Table 2** The characteristics of cMIND dietary components.

| Components | Score | | |
| --- | --- | --- | --- |
|  | 0, n (%) | 0.5, n (%) | 1, n (%) |
| Types of staple food | 13496 (96.2) |  | 535 (3.8) |
| Amount of staple food | 7564 (53.9) |  | 6467 (46.1) |
| Fresh fruit | 7747 (55.2) | 3356 (23.9) | 2928 (20.9) |
| Fresh vegetables | 1623 (11.6) | 3534 (25.2) | 8874 (63.2) |
| Cooking oil | 1462 (10.4) |  | 12569 (89.6) |
| Mushroom or algae | 11485 (81.9) | 2178 (15.5) | 368 (2.6) |
| Fish | 4771 (34.0) | 2738 (19.5) | 6522 (46.5) |
| Food made from beans | 6871 (49.0) | 5518 (39.3) | 1642 (11.7) |
| Nut | 11546 (82.3) | 1669 (11.9) | 816 (5.8) |
| Garlic | 7879 (55.8) | 3442 (24.5) | 2760 (19.7) |
| Tea | 11820 (84.2) | 910 (6.5) | 1301 (9.3) |
| White sugar or candy | 4152 (29.6) | 1677 (12.0) | 8202 (58.5) |
