# Supplementary material for: Associations between cMIND diet, mold exposure, and visual impairment among older adults in China: a national cross-sectional study
Source: Front Nutr. 2026 Jul 6;13:1851210. doi: 10.3389/fnut.2026.1851210 (PMC13381192; doi:10.3389/fnut.2026.1851210)
Supplement: Supplementary file 3 [file Table_3.docx]

**Supplementary Table 3** Association of cMIND diet with visual impairment, stratified by categorical variables.

|  | OR (95%CI) | P-value | P for interaction |
| --- | --- | --- | --- |
| Sex |  |  | 0.811 |
| Female | 0.91 (0.87, 0.94) | <0.001 |  |
| Male | 0.89 (0.85, 0.94) | <0.001 |  |
| Area of residence |  |  | 0.048 |
| Urban | 0.83 (0.78, 0.89) | <0.001 |  |
| Rural | 0.93 (0.90, 0.97) | <0.001 |  |
| Marital status |  |  | 0.171 |
| Have no spouse | 0.90 (0.87, 0.94) | <0.001 |  |
| Have a spouse | 0.90 (0.85, 0.95) | <0.001 |  |
| Education level |  |  | 0.424 |
| 0 year | 0.90 (0.86, 0.94) | <0.001 |  |
| 1-6 years | 0.93 (0.88, 0.99) | 0.023 |  |
| ≥ 7 years | 0.89 (0.82, 0.97) | 0.005 |  |
| Smoking status |  |  | 0.247 |
| No | 0.90 (0.87, 0.93) | <0.001 |  |
| Yes | 0.93 (0.86, 1.02) | 0.117 |  |
| Alcohol consumption |  |  | 0.012 |
| No | 0.89 (0.86, 0.93) | <0.001 |  |
| Yes | 0.98 (0.90, 1.08) | 0.734 |  |
| Physical activity |  |  | 0.966 |
| No | 0.91 (0.87, 0.94) | <0.001 |  |
| Yes | 0.91 (0.85, 0.97) | 0.003 |  |
| Hypertension |  |  | 0.310 |
| No | 0.89 (0.86, 0.93) | <0.001 |  |
| Yes | 0.93 (0.88, 0.98) | 0.004 |  |
| Diabetes |  |  | 0.629 |
| No | 0.91 (0.88, 0.94) | <0.001 |  |
| Yes | 0.91 (0.82, 1.01) | 0.080 |  |
| Heart disease |  |  | 0.735 |
| No | 0.91 (0.88, 0.94) | <0.001 |  |
| Yes | 0.88 (0.82, 0.95) | 0.001 |  |
| Dementia |  |  | 0.428 |
| No | 0.91 (0.88, 0.94) | <0.001 |  |
| Yes | 0.87 (0.67, 1.13) | 0.300 |  |

Abbreviation: OR: Odds ratios, CI: Confidence intervals.
