# Supplementary material for: Associations between cMIND diet, mold exposure, and visual impairment among older adults in China: a national cross-sectional study
Source: Front Nutr. 2026 Jul 6;13:1851210. doi: 10.3389/fnut.2026.1851210 (PMC13381192; doi:10.3389/fnut.2026.1851210)
Supplement: Supplementary file 4 [file Table_4.docx]

**Supplementary Table 4** Association of mold exposure with visual impairment, stratified by categorical variables.

|  | OR (95%CI) | P-value | P for interaction |
| --- | --- | --- | --- |
| Sex |  |  | 0.557 |
| Female | 1.15 (0.97, 1.36) | 0.112 |  |
| Male | 1.24 (1.02, 1.52) | 0.035 |  |
| Area of residence |  |  | <0.001 |
| Urban | 1.88 (1.37, 2.59) | <0.001 |  |
| Rural | 1.07 (0.93, 1.23) | 0.371 |  |
| Marital status |  |  | 0.056 |
| Have no spouse | 1.31 (1.11, 1.54) | 0.001 |  |
| Have a spouse | 1.01 (0.82, 1.26) | 0.904 |  |
| Education level |  |  | <0.001 |
| 0 year | 1.03 (0.87, 1.21) | 0.734 |  |
| 1-6 years | 1.23 (0.96, 1.58) | 0.107 |  |
| ≥ 7 years | 2.08 (1.45, 3.00) | <0.001 |  |
| Smoking status |  |  | 0.117 |
| No | 1.24 (1.08, 1.43) | 0.003 |  |
| Yes | 0.92 (0.65, 1.29) | 0.614 |  |
| Alcohol consumption |  |  | 0.061 |
| No | 1.24 (1.08, 1.42) | 0.003 |  |
| Yes | 0.88 (0.60, 1.30) | 0.528 |  |
| Physical activity |  |  | 0.612 |
| No | 1.17 (1.01, 1.36) | 0.035 |  |
| Yes | 1.23 (0.94, 1.62) | 0.137 |  |
| Hypertension |  |  | 0.390 |
| No | 1.25 (1.05, 1.48) | 0.012 |  |
| Yes | 1.10 (0.90, 1.34) | 0.365 |  |
| Diabetes |  |  | 0.010 |
| No | 1.12 (0.97, 1.28) | 0.115 |  |
| Yes | 1.79 (1.19, 2.67) | 0.005 |  |
| Heart disease |  |  | 0.350 |
| No | 1.15 (1.00, 1.33) | 0.054 |  |
| Yes | 1.32 (0.96, 1.82) | 0.084 |  |
| Dementia |  |  | 0.496 |
| No | 1.18 (1.03, 1.34) | 0.015 |  |
| Yes | 1.65 (0.66, 4.13) | 0.282 |  |

Abbreviation: OR: Odds ratios, CI: Confidence intervals.
