# Supplementary material for: Associations between cMIND diet, mold exposure, and visual impairment among older adults in China: a national cross-sectional study
Source: Front Nutr. 2026 Jul 6;13:1851210. doi: 10.3389/fnut.2026.1851210 (PMC13381192; doi:10.3389/fnut.2026.1851210)
Supplement: Supplementary file 5 [file Table_5.docx]

**Supplementary Table 5** Stratified joint effects of cMIND diet and mold exposure on visual impairment by area of residence.

| cMIND diet, score | Mold exposure | Urban | | Rural | |
| --- | --- | --- | --- | --- | --- |
|  |  | OR (95%CI) | P-value | OR (95%CI) | P-value |
| 0-4 |  |  |  |  |  |
|  | Had no mold exposure | 2.20 (1.65, 2.92) | <0.001 | 1.38 (1.17, 1.63) | <0.001 |
|  | Had mold exposure | 2.57 (1.47, 4.51) | <0.001 | 1.44 (1.15, 1.79) | 0.001 |
| 4.5-5.5 |  |  |  |  |  |
|  | Had no mold exposure | 1.57 (1.22, 2.02) | <0.001 | 1.23 (1.04, 1.47) | 0.017 |
|  | Had mold exposure | 3.16 (1.83, 5.43) | <0.001 | 1.33 (1.01, 1.75) | 0.046 |
| 6-12 |  |  |  |  |  |
|  | Had no mold exposure | 1.00 | - | 1.00 | - |
|  | Had mold exposure | 2.21 (1.32, 3.70) | 0.003 | 0.94 (0.60, 1.46) | 0.776 |

Adjusted for age, sex, ethnicity, marital status, education level, smoking status, alcohol consumption, physical activity, hypertension, diabetes, heart disease, and dementia.
