# Supplementary material for: Associations between cMIND diet, mold exposure, and visual impairment among older adults in China: a national cross-sectional study
Source: Front Nutr. 2026 Jul 6;13:1851210. doi: 10.3389/fnut.2026.1851210 (PMC13381192; doi:10.3389/fnut.2026.1851210)
Supplement: Supplementary file 6 [file Table_6.docx]

**Supplementary Table 6** Stratified joint effects of cMIND diet and mold exposure on visual impairment by alcohol consumption.

| cMIND diet, score | Mold exposure | Alcohol consumption | | | |
| --- | --- | --- | --- | --- | --- |
|  |  | No | | Yes | |
|  |  | OR (95%CI) | P-value | OR (95%CI) | P-value |
| 0-4 |  |  |  |  |  |
|  | Had no mold exposure | 1.65 (1.42, 1.92) | <0.001 | 1.22 (0.82, 1.82) | 0.319 |
|  | Had mold exposure | 1.87 (1.51, 2.32) | <0.001 | 0.69 (0.36, 1.30) | 0.250 |
| 4.5-5.5 |  |  |  |  |  |
|  | Had no mold exposure | 1.39 (1.19, 1.62) | <0.001 | 1.30 (0.89, 1.89) | 0.170 |
|  | Had mold exposure | 1.62 (1.25, 2.10) | <0.001 | 2.12 (1.10, 4.09) | 0.025 |
| 6-12 |  |  |  |  |  |
|  | Had no mold exposure | 1.00 | - | 1.00 | - |
|  | Had mold exposure | 1.53 (1.06, 2.20) | 0.023 | 0.90 (0.41, 1.98) | 0.787 |

Adjusted for age, sex, area of residence, ethnicity, marital status, education level, smoking status, physical activity, hypertension, diabetes, heart disease, and dementia.
