# Supplementary material for: Associations between cMIND diet, mold exposure, and visual impairment among older adults in China: a national cross-sectional study
Source: Front Nutr. 2026 Jul 6;13:1851210. doi: 10.3389/fnut.2026.1851210 (PMC13381192; doi:10.3389/fnut.2026.1851210)
Supplement: Supplementary file 7 [file Table_7.docx]

**Supplementary Table 7** Stratified joint effects of cMIND diet and mold exposure on visual impairment by education level.

| cMIND diet, score | Mold exposure | Education level | | | | | |
| --- | --- | --- | --- | --- | --- | --- | --- |
|  |  | 0 years | | 1-6 years | | ≥ 7 years | |
|  |  | OR (95%CI) | P-value | OR (95%CI) | P-value | OR (95%CI) | P-value |
| 0-4 |  |  |  |  |  |  |  |
|  | Had no mold exposure | 1.61  (1.32, 1.97) | <0.001 | 1.28  (0.99, 1.64) | 0.060 | 1.83  (1.26, 2.65) | 0.002 |
|  | Had mold exposure | 1.48  (1.14, 1.93) | 0.003 | 1.77  (1.21, 2.58) | 0.003 | 2.86  (1.45, 5.62) | 0.002 |
| 4.5-5.5 |  |  |  |  |  |  |  |
|  | Had no mold exposure | 1.22  (0.99, 1.50) | 0.067 | 1.53  (1.20, 1.95) | <0.001 | 1.48  (1.07, 2.03) | 0.017 |
|  | Had mold exposure | 1.37  (0.99, 1.91) | 0.061 | 1.80  (1.17, 2.78) | 0.008 | 2.51  (1.32, 4.77) | 0.005 |
| 6-12 |  |  |  |  |  |  |  |
|  | Had no mold exposure | 1.00 | - | 1.00 | - | 1.00 | - |
|  | Had mold exposure | 1.08  (0.64, 1.82) | 0.771 | 0.76  (0.37, 1.57) | 0.460 | 2.77  (1.60, 4.79) | <0.001 |

Adjusted for age, sex, area of residence, ethnicity, marital status, education level, smoking status, alcohol consumption, physical activity, hypertension, diabetes, heart disease, and dementia.
