# Supplementary material for: Associations between cMIND diet, mold exposure, and visual impairment among older adults in China: a national cross-sectional study
Source: Front Nutr. 2026 Jul 6;13:1851210. doi: 10.3389/fnut.2026.1851210 (PMC13381192; doi:10.3389/fnut.2026.1851210)
Supplement: Supplementary file 8 [file Table_8.docx]

**Supplementary Table 8** Stratified joint effects of cMIND diet and mold exposure on visual impairment by diabetes.

| cMIND diet, score | Mold exposure | Diabetes | | | |
| --- | --- | --- | --- | --- | --- |
|  |  | No | | Yes | |
|  |  | OR (95%CI) | P-value | OR (95%CI) | P-value |
| 0-4 |  |  |  |  |  |
|  | Had no mold exposure | 1.60 (1.38, 1.86) | <0.001 | 1.26 (0.79, 2.01) | 0.326 |
|  | Had mold exposure | 1.66 (1.34, 2.05) | <0.001 | 1.56 (0.81, 3.02) | 0.187 |
| 4.5-5.5 |  |  |  |  |  |
|  | Had no mold exposure | 1.38 (1.19, 1.61) | <0.001 | 1.21 (0.80, 1.83) | 0.364 |
|  | Had mold exposure | 1.52 (1.17, 1.97) | 0.002 | 2.98 (1.50, 5.92) | 0.002 |
| 6-12 |  |  |  |  |  |
|  | Had no mold exposure | 1.00 | - | 1.00 | - |
|  | Had mold exposure | 1.26 (0.87, 1.82) | 0.224 | 1.86 (0.89, 3.92) | 0.101 |

Adjusted for age, sex, area of residence, ethnicity, marital status, education level, smoking status, physical activity, hypertension, heart disease, and dementia.
