# Supplementary material for: Associations between cMIND diet, mold exposure, and visual impairment among older adults in China: a national cross-sectional study
Source: Front Nutr. 2026 Jul 6;13:1851210. doi: 10.3389/fnut.2026.1851210 (PMC13381192; doi:10.3389/fnut.2026.1851210)
Supplement: Supplementary file 10 [file Table_10.docx]

**Supplementary Table 10** Multiple imputation models on the association of mold exposure with visual impairment.

| Model | Had no mold exposure | Had mold exposure | P-value |
| --- | --- | --- | --- |
|  | OR (95%CI) | |  |
| Model 1 | Reference | 1.26 (1.15, 1.39) | <0.001 |
| Model 2 | Reference | 1.23 (1.10, 1.37) | <0.001 |
| Model 3 | Reference | 1.20 (1.08, 1.34) | <0.001 |

Abbreviation: OR: Odds ratios, CI: Confidence intervals.

Model 1 was unadjusted.

Model 2 was adjusted for age, sex, area of residence, ethnicity, marital status, and education level.

Model 3 was adjusted for age, sex, area of residence, ethnicity, marital status, education level, smoking status, alcohol consumption, physical activity, hypertension, diabetes, heart disease, and dementia.
