# Supplementary material for: Associations between cMIND diet, mold exposure, and visual impairment among older adults in China: a national cross-sectional study
Source: Front Nutr. 2026 Jul 6;13:1851210. doi: 10.3389/fnut.2026.1851210 (PMC13381192; doi:10.3389/fnut.2026.1851210)
Supplement: Supplementary file 11 [file Table_11.docx]

**Supplementary Table 11** Associations of cMIND diet with visual impairment, after excluding participants who reported hypertension, diabetes, and heart disease.

| Variables | Model 1 | | Model 2 | | Model 3 | |
| --- | --- | --- | --- | --- | --- | --- |
|  | OR (95%CI) | P-value | OR (95%CI) | P-value | OR (95%CI) | P-value |
| cMIND diet was used as a continuous variable | 0.74  (0.72, 0.77) | <0.001 | 0.85  (0.82, 0.89) | <0.001 | 0.89  (0.85, 0.93) | <0.001 |
| cMIND diet was used as a categorical variable (vs. Lower (0-4)) | - | - | - | - | - | - |
| Medium (4.5-5.5) | 0.56  (0.50, 0.62) | <0.001 | 0.75  (0.66, 0.86) | <0.001 | 0.83  (0.72, 0.96) | <0.001 |
| High (6-12) | 0.34  (0.30, 0.39) | <0.001 | 0.53  (0.45, 0.63) | <0.001 | 0.59  (0.49, 0.71) | <0.001 |

Abbreviation: OR: Odds ratios, CI: Confidence intervals.

Model 1 was unadjusted.

Model 2 was adjusted for age, sex, area of residence, ethnicity, marital status, and education level.

Model 3 was adjusted for age, sex, area of residence, ethnicity, marital status, education level, smoking status, alcohol consumption, physical activity, hypertension, diabetes, heart disease, and dementia.
